# Cytoreductive surgery (CRS) plus hyperthermic intraperitoneal chemotherapy (HIPEC) vs CRS alone for treatment of endometrial cancer with peritoneal metastases: a multi-institutional study from PSOGI and BIG RENAPE groups

**DOI:** 10.1186/s12893-021-01449-z

**Published:** 2022-01-07

**Authors:** Manuel Gomes David, Naoual Bakrin, Julia Salleron, Marie Christine Kaminsky, Jean Marc Bereder, Jean Jacques Tuech, Kuno Lehmann, Sanket Mehta, Olivier Glehen, Frédéric Marchal

**Affiliations:** 1 Département de Chirurgie Oncologique, Institut de Cancérologie de Lorraine, Université de Lorraine, 6 Avenue de Bourgogne, 54519 Vandoeuvre-lès-Nancy, France; 2 Service de Chirurgie Digestive et Endocrinienne, Hôpital Lyon Sud, Hospices Civils de Lyon, Lyon, France; 3 Institut de Cancérologie de Lorraine, Cellule Data Biostatistiques, Université de Lorraine, 54519 Vandœuvre-lès-Nancy, France; 4 Département d’oncologie, Institut de Cancérologie de Lorraine, Université de Lorraine, 54519 Vandœuvre-lès-Nancy, France; 5 Nice University Hospital, Nice, France; 6 Rouen University Hospital, Rouen, France; 7 Zurich University Hospital, Zurich, Switzerland; 8 Saiffee Hospital, Mumbai, India; 9 CRAN, UMR 7039, CNRS, Université de Lorraine, Boulevard des Aiguillettes, 54506 Vandoeuvre-les-Nancy, France

**Keywords:** HIPEC, Endometrial cancer, Carcinomatosis, Cytoreductive surgery, Peritoneal metastasis

## Abstract

**Objective:**

To investigate the benefit of cytoreductive surgery (CRS) combined with hyperthermic intraperitoneal chemotherapy (HIPEC) for the treatment of endometrial peritoneal carcinomatosis compared to CRS alone.

**Methods:**

We conducted a retrospective multicentre study of patients from experienced centres in treating peritoneal malignancies from 2002 to 2015. Patients who underwent surgery for peritoneal evolution of endometrial cancer (EC) were included. Two groups of 30 women were matched and compared: “CRS + HIPEC” which used HIPEC after CRS, and “CRS only” which did not use HIPEC. We analysed clinical, pathologic and treatment data for patients with peritoneal metastases from EC. The outcome measures were morbidity, overall survival (OS), and progression-free survival (PFS).

**Results:**

In “CRS plus HIPEC” group, 96.7% of women were treated for recurrence, while in “CRS only” 83.3 were treated for primary disease. There was no significant difference between Peritoneal Carcinomatosis Index at laparotomy or Completeness of Cytoreduction score. Grade III and IV complications rates did not significantly differ between “CRS plus HIPEC” group and “CRS only” group (20.7% vs 20.7%, p = 0.739). Survival analysis showed no statistical difference between both groups. Median OS time was 19.2 months in “CRS plus HIPEC” group and 29.7 months in “CRS only” group (p = 0.606). Median PFS survival time was 10.7 months in “CRS plus HIPEC” group and 13.1 months in “CRS only” group (p = 0.511).

**Conclusion:**

The use of HIPEC combined to CRS did not have any significance as regard the DFS and OS over CRS alone in patients with primary or recurrent peritoneal metastasis of endometrial cancer.

## Introduction

Endometrial cancer (EC) is the most common gynecologic malignancy in developed countries. Because of early vaginal bleeding, most diseases are diagnosed early stages and result in a favourable prognosis [1]. Although the overall 5-years survival reaches 95% for early detected cancers (75 to 80% of cases), nearly 10% to 15% of women with early-stage disease (International Federation of Gynaecology and Obstetrics (FIGO) stage I and II) develop recurrences [2, 3]. Advanced-stage endometrial cancers represent only 15% of newly diagnosed cases but are associated with a poor prognosis. The 5-year survival rates drop in women with regional (49 to 66%) or distant spread (from 20 to 25%), resulting in median survival of less than 1 year in cases of disseminated disease confined to the peritoneum [4, 5].

Management of women with primitive or recurrent peritoneal dissemination remains heterogeneous. Prior treatment history and patient’s performance status is considered and involve surgical resection, systemic chemotherapy, brachytherapy, radiation or hormone therapy. The benefit of optimal surgical cytoreduction (CRS) in the management of advanced ovarian cancer has been established by multiple studies. Its role in management of advanced or recurrent endometrial cancer remains uncertain but significant survival benefit can be achieved with optimal resection [3, 6, 7]. CRS combined with hyperthermic intraperitoneal chemotherapy (HIPEC) has shown promising results in patients with primary peritoneal tumors such as pseudomyxoma peritonei, peritoneal mesothelioma or peritoneal metastases from colorectal, gastric, and ovarian cancer [8–12].

Few series with a small number of patients have been reported of CRS with HIPEC for treatment of EC with promising outcomes and well-tolerated procedures [4, 13–17].

We compared two series of patients who had peritoneal metastases (PM) of EC, one treated with CRS in an experienced centre and the other treated with CRS and HIPEC in ten experienced centres from the PSOGI and BIG RENAPE groups [9].

The objective of the study was to investigate the benefit in terms of disease-free and overall survival times of CRS combined with HIPEC compared to CRS alone for the treatment of endometrial PM.

## Materials and methods

### Patient population

From a multi-center international database (collaborative database of Peritoneal Surface Oncology Group International (PSOGI) and BIG-RENAPE working groups [9]), the “CRS plus HIPEC” group represented patients with PM treated with CRS and HIPEC. The “CRS alone” patients with PM treated with CRS but without HIPEC were retrieved from hospital database of the Institut de Cancérologie de Lorraine. Ethics approval was obtained from the participating institutions through their institutional review boards or through the chairpersons of their ethics committees.

The inclusion criteria were patients with primary or advanced peritoneal carcinomatosis of endometrial origin, giving consent to the procedure and without contraindications either to CRS alone or HIPEC. Patients with pre-operative extra-abdominal metastasis, unresectable disease or lack of fitness for the procedure were excluded.

A total of 44 patients in the “CRS plus HIPEC” group and 90 patients in the “CRS alone” fulfilled inclusion and exclusion criteria. In order to control the potential confounding factors, patients of “CRS plus HIPEC” and “CRS alone” groups were 1:1 matched by the global optimal algorithm [18] based on propensity score. The exact matching was performed on three criteria: age at diagnosis (± 10 years), histological type (endometrioid vs adenocarcinoma vs other carcinosarcoma), and year of surgery (± 5 ans); the propensity score was computed by a multivariate logistic regression with group as dependant parameter and all patients and clinical characteristics as independent parameters.

The main clinical data were collected retrospectively from patients treated for peritoneal carcinomatosis. Age, histological type, tumor histology, peritoneal cancer index (PCI), surgical procedure, HIPEC techniques and drugs, completeness of cytoreduction (CC) score, data regarding primary treatment, chemotherapy, postoperative complications according to the common terminology criteria for adverse events (CTCAE) v3.0 of the National Institute of Health and complete follow-up information were collected. Staging was performed on imaging data, including computed tomography (CT), magnetic resonance imaging, positron emission CT or laparoscopic exploration for resectability evaluation. Approval of treatment were established at multidisciplinary meetings.

All surgical explorations and procedures were under the direction a senior surgeon. All patients were judged to be completely resectable during surgical exploration. The extent of carcinomatosis was assessed using the Peritoneal Cancer Index (PCI), obtaining a score between 0 and 39 [19]. Surgery was performed in order to obtain a complete resection of all visible tumor nodules. Peritonectomy procedures were performed when the peritoneal surfaces were macroscopically affected. After completion of the surgical cytoreduction, the Completeness of Cytoreduction Score (CC-S) was evaluated by the surgeon before HIPEC perfusion and was classified as follows: CC-0 = no macroscopic residual cancer, CC-1 = residual nodules < 2.5 mm, CC-2 = residual nodule between 2.5 and 25 mm, CC-3 = residual nodule > 25 mm.

HIPEC was delivered at the end of surgery according to centers preferences and the technic previously described [20]. The intraperitoneal chemotherapy protocol used cisplatin, doxorubicin or mitomycin. The mixture was placed in contact with the peritoneal cavity at a dose of 2 l/m^2^ of body surface for 60 to 90 min at a controlled temperature between 41 and 43 °C.

The overall survival (OS) was evaluated from the date of surgery to the date of death or last follow-up, and reported at 3 and 5 years. The progression free survival (PFS) was evaluated from the date of surgery to the date of documented disease progression or recurrence assessed on cross-sectional imaging.

### Statistical analysis

Quantitative parameters were described as mean and standard deviation or by median and interquartile range (IQR) and qualitative parameters as frequency and percentage. Normality of the distribution was assessed by Shapiro–Wilk test. Patients’ characteristics at surgery were compared between the two groups with paired sample Student t-test or paired sample Wilcoxon test or Mac Nemar test in order to take into account the matching and paired differences were computed.

OS and PFS were described by the Kaplan Meier method and compared by univariate Cox proportional hazards regression model using a robust sandwich‐type variance estimator for the clustering within matched groups. Results were adjusted on the remaining unbalanced characteristics between the two groups by a multivariate Cox proportional hazards regression model. Results were expressed as hazard ratio (HR) and 95% confidence interval (95% CI) with “CRS only” group as reference.

The percentage of Grade 3 and 4 complications was compared with Mac Nemar test.

Significance level was set at 5%. The analyses were performed with SAS software version 9.4 (SAS Institute Inc., Cary, NC, USA).

### Ethics

This research was performed in accordance with the Declaration of Helsinki and was approved by an appropriate ethics committee. The French National Data Protection Authority approved this study: Commission Nationale de l'Informatique et des Libertés de France—INDS n° 1510270220). A written consent has been obtained for all participants.

## Results

From 2002 to 2015, 44 women underwent CRS plus HIPEC and 30 women underwent CRS only for treatment of peritoneal metastases of endometrial cancer. After application of the matching criteria, 30 women were included in the “CRS plus HIPEC” group and 30 women in the “CRS only” group. The 30 women who received CRS plus HIPEC were treated in ten experienced centres.

Table 1 summarizes the women’s characteristics. Women in “CRS plus HIPEC” group were younger compared with the “CRS only” group (estimated mean difference of 2.5 years, p = 0.004). The mean duration of tumor progression between diagnosis of peritoneal involvement and the surgical procedure was months shorter in the “CRS only” group (mean difference of 3.9 months, IQR from − 0.1 to 8.5, p = 0.001): 5 patients (16.7%) of “CRS only” group were treated for peritoneal recurrences whereas 1 patient (3.3%) of “CRS plus HIPEC” was treated with primary surgery. There was no significant difference between PCI at laparotomy or CCS between the two groups.

**Table 1:** Patients’ characteristics at surgery in the two groups

	CRS only *n* = *30*	CRS + HIPEC *n* = *30*	Paired differences	p
Age (years)	63.9 (60.5;67.2)	60.9 (58.9;62.9)	− 2.5 (− 4.5;− 0.5)	0.004^‡^
Tumor histology				
Endometrioid adenocarcinoma	27 (90.0%)	27 (90.0%)	–	1^‡^
Carcinosarcoma	1 (3.3%)	1 (3.3%)		
Other	2 (6.7%)	2 (6.7%)		
Surgery				< 0.001
Primary	25 (83.3%)	1 (3.3%)	− 80.0% (− 97.0%;− 63.0%)	
Recurrence	5 (16.7%)	29 (96.7%)	–	
Months since diagnosis	2.1 (5.4;19.5)^*^	6.8 (5.1;9.2)^*^	3.9 (− 0.1;8.5)^*^	0.001
Systemic chemotherapy^◊^				
Neoadjuvant	3 (10.0%)	11 (44.7%)	30.7% (11.1%;50.4%)	0.011
Adjuvant	27 (90.0%)	16 (59.4%)	–	
PCI	10.0 (5.6;14.4)	9.9 (7.5;12.2]	− 0.1 (− 4.3;4.1)	0.702
CC score^∆^				
CC-0	21 (72.4%)	23 (79.3%)	6.9% (− 6.1%;29.9%)	0.763
CC-1 or CC-2	8 (25.6%)	6 (20.7%)	–	
Surgery duration (hours)	4.0 (3.3;4.6)	6.1 (5.3;6.9)	2.1 (1.0;3.1)	0.006

Results presented as Mean and 95% confidence interval and frequency and percentage unless otherwise specified

PCI: Peritoneal Cancer Index; Completeness of Cytoreduction Score (CC-S): CC-0 = no macroscopic residual cancer, CC-1 = residual nodules < 2.5 mm, CC-2 = residual nodule between 2.5 and 25 mm

^*^Median and interquartile range

^‡^Matching criteria

^◊^3 missing data

^∆^2 missing data

For women who underwent CRS plus HIPEC, when surgical procedures ended, HIPEC was administered with a single drug: Cisplatin for 75.9%, Mitomycin for 17.2% and Oxaliplatin for 6.9% at a temperature of 42 to 43 °C.

There was no post-operative death on both groups. Grade III and IV complications (Table 2) occurred for 6 patients in each group. One woman (3.3%) in each group experienced abdominal haemorrhage and required blood transfusion. The most frequent complication was gastrointestinal complication (overall 11.8% of women).

**Table 2:** Procedure complications

	CRS only *n* = *30*	CRS + HIPEC *n* = *30*	p
Grade 3 and 4 complications	6 (20.7%)	6 (20.7%)	0.739
Abdominal haemorrhage	1 (3.3%)	1 (3.3%)	–
Cardiac	1 (3.3%)	1 (3.3%)	–
Gastrointestinal	6 (20.0%)	1 (3.3%)	–
Others	0	3 (10.3%)	–

**Fig. 1:**
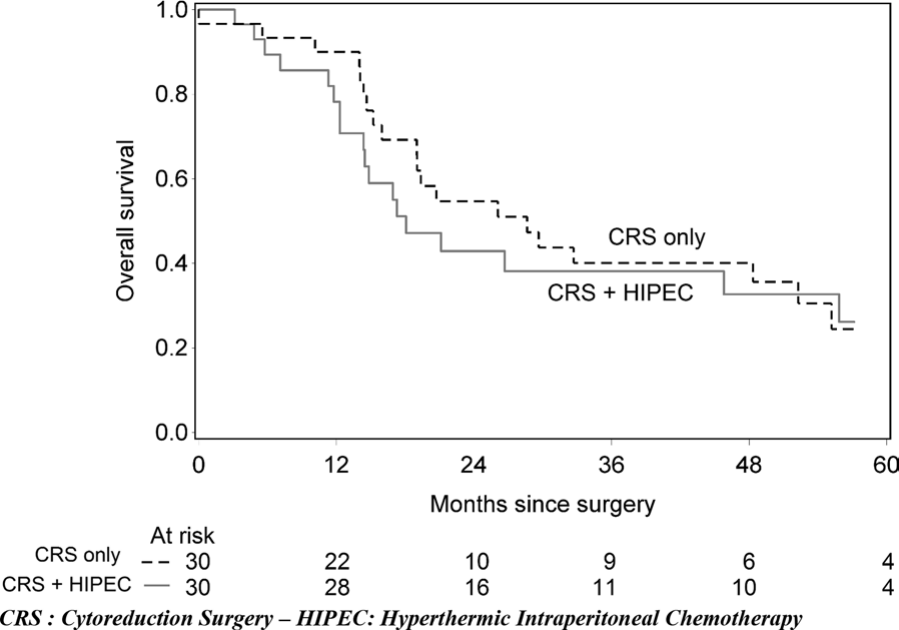
Overall survival from the surgery in “CRS plus HIPEC” and “CRS only” groups

**Fig. 2:**
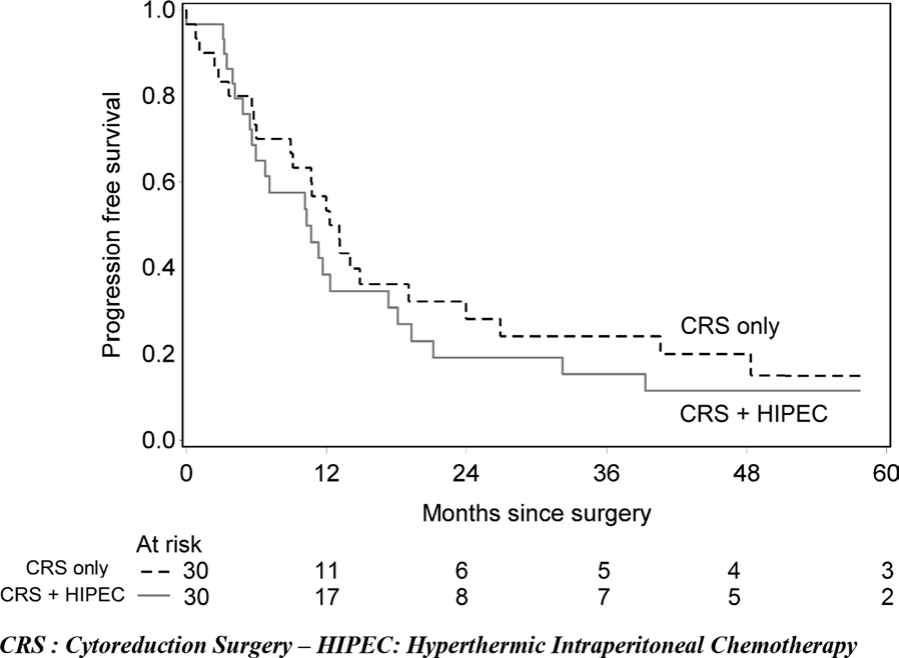
Progression-free survival from the surgery in “CRS plus HIPEC” and “CRS only” groups

The median follow-up time since surgery was 50 months (IQR from 19 to 94) for the 22 patients alive at the end of follow-up time. On the all population, it was 19 months (14–50): 17.1 months (IQR from 12 to 46) for “CRS plus HIPEC” group and 26 months (IQR from 15 to 52) for “CRS only” (p = 0.172). In univariate analysis, overall survival was not significantly different (HR 1.18, 95% CI [0.62;2.26], p = 0.606). Overall median survival time was 19.2 months, 95% [12.5;57.1] in “CRS plus HIPEC” group and 29.7 months, 95% CI [17.8;53.5] in “CRS only” group. At 12 months, overall survival was 81.9%, 95% CI [61.9%;92.1%] for “CRS plus HIPEC” group and 93.3%, 95% CI [75.9%;98.3%] for CRS only (Fig. 1). Progression free survival was also not statistically different (HR 1.22, 95% CI [0.67;2.22], p = 0.511). At 12 months, PFS was 42.2%%, 95% CI [23.7%;59.6%] for “CRS plus HIPEC” group and 56.6%, 95% CI [37.3%;72.1%] for CRS only (Fig. 2). Progression-free median survival time was 10.7 months, 95% CI [5.9;18.1] in “CRS plus HIPEC” group and 13.1 months, 95% CI [9.1;24.0] in “CRS only” group. After adjustment on the time from diagnosis of peritoneal involvement to the surgical procedure, age at surgery and chemotherapy, the differences between the two groups remained not statistically significant for OS (HR 1.65 [0.76;3.60], p = 0.207) nor PFS (HR 1.427, 95% CI [0.71;2.85], p = 0.324).

## Discussion

Despite an initial favorable prognosis, endometrial cancer recurrences range from 2 to 15% in patients with an early-stage disease (stage I and II) to 50% in patients with an advanced-stage disease (stage III and IV) [21, 22]. Fifty to 70% of recurrences occur within 2 years after primary management [23]. Association of CRS and platinum-based systemic chemotherapy is the standard strategy for selected patients with synchronous and metachronous peritoneal metastasis. However, there is a need for improving the outcomes of patients with endometrial cancer peritoneal metastasis. Combination of CRS and HIPEC has been proposed, with promising outcomes. In a systematic review of eight studies, Tempfer et al. demonstrate that CRS and HIPEC in this indication is feasible and has an acceptable morbidity and mortality. Still, these data do not prove an independent effect of HIPEC [24]. It is also possible that the same good results would have been achieved with CRS and systemic chemotherapy alone. Therefore, comparative clinical trials are needed to prove the therapeutic value of HIPEC in addition to CRS in this indication. In this retrospective study, we compared two series of 30 women undergoing either CRS or CRS and HIPEC for peritoneal metastasis of endometrial cancer. In the two groups, a CC-0 resection was achieved in 73.3% of patients and there was no treatment-associated mortality. No statistical difference was observed in grade 3 and 4 adverse events (20.7 vs 20.7%, p = 0.739). Moreover, there was no difference in median disease-free and overall survival times between the two groups.

Nowadays, systemic chemotherapy based on a combination of Doxorubicin and Cisplatin is the standard therapy for EC recurrences. Unfortunately, the median survival is above 1 year [25, 26]. Fleming et al., in a Phase III randomized trial, observed an increase of 20% in overall survival when Doxorubicin and Cisplatin are combined with Paclitaxel (12.3 vs 15.3 months). However, this combination has not adopted because of the much higher toxicity [27]. There was no difference between the two group in grade III and IV complication rates in our study (20.7 to 20.7% respectively). Despite the high rate of complications, the overall and progression-free survival times observed for patients with CRS alone or CRS plus HIPEC, 26.0 and 12.0 months respectively, suggests that a surgical approach securing a lack of postoperative residue is legitimate for selected patients. There is a need to investigate the role of cytoreductive surgery in management of advanced endometrial cancer compared to radiation or chemotherapy alone. Data available on literature, suggest that CRS improves overall survival of patients compared to radiation or chemotherapy alone but, its role is still not well established [28]. In a recent meta-analysis, Barlin et al. observed that complete cytoreduction and adjuvant radiation were positively associated with survival, whereas adjuvant chemotherapy was associated with a decreased survival [29]. Navarro-Barrios et al. found four significative criteria for optimal patient selection: primary cytoreductive surgery without preoperative chemotherapy, limited surgical maneuvers, use of cisplatin and no lymph node involvement [30]. However, these studies are limited by their retrospective nature. Additional randomized studies are needed comparing both survival and treatment grade III and IV complication rates and their acceptability between a surgical and a medical therapeutic approach.

This study is, to our knowledge, the first to assess the therapeutic value of HIPEC in addition to CRS in patients with peritoneal metastasis of endometrial cancer. The clinical characteristics and outcomes in both groups were consistent with other studies describing the use of HIPEC in addition to CRS (Table 3). Moreover, our study has a large sample size; to our knowledge it is currently the only study that combines such a large size with good control for potential bias thanks to propensity score matching. Indeed, there was no difference between the two series comparing the most significant prognostic factors in endometrial cancer patients such as advanced age of patients, histological types, initial PCI or residual disease. However, there was a difference between the two groups in terms of the time-to-treatment duration. This may be explained by the fact that almost all patients who had received HIPEC had surgical management for peritoneal recurrence compared to only five women in the “CRS only” group. The main difference between the two groups was disease phase. “CRS only” women mainly experience initial advanced disease whereas almost all patients who had received HIPEC had surgical management for peritoneal recurrence. Women who initially present with advanced disease have a 5-year OS rate of 16%. These women also have higher rates of recurrence. Peritoneal recurrence occurs in 10 to 15% of endometrial patients. The 5-year overall survival is reduced to 17% for extrapelvic recurrences. After optimal CRS, an overall survival after recurrence (OSAR) of 16 to 29 months could be achieved [31, 32].

**Table 3:** Clinical characteristics and outcomes of women undergoing cytoreductive surgery and HIPEC

Author	Number of patients	Age (years, mean)	Time since initial treatment (months)	PCI	CRS caracreristics	Morbidity	Mortality	PFS (months, median; range)	OS (months, median)
Helm [17]	5	61	47 (mean; range 29–66)	–	CCO: 60.0% CC1: 20.0% CC2: 20.0%	Grade 3: 0% Grade 4: 60.0%	0/5	7 (0–32)	28
Bakrin [4]	5	59.6	47.5 (mean; range 10–120)	7 (median; range 5–18)	CC0: 100%	Grade 3: 40.0% Grade 4: 32.0%	0/5	10 (2–39)	16
Santeufemia [16]	1	70	120	–	–	0%	0/1	12	12
Delotte [15]	13	66.5	18.5 (median; range 0–53)	12 (median; range 3–24)	CCO: 61.5% CC1: 23.1% CC2: 15.4%	–	0/13	11 (2–124)	19.4
Abu-Zaid [14]	6	55.5	9 (mean; range 1–18)	19 (mean; range 9–26)	CC0: 83.3% CC1: 16.7%	Grade 3: 0% Grade 4: 33.3%	0/6	13 (3–35)	–
Cornali [13]	33	57.7	17.5 (median; range 6–36)	15 (median; range 5–35)	CCO: 66.7% CC1: 21.2% CC2: 12.1%	Grade 3: 15.2% Grade 4: 3.0%	1/33	18	33.1
Navarro-Barrios [30]	43	62.0	–	12 (median, range 7–19)	CCO: 95% CC1 or 2: 5%	Grade 3: 0% Grade 4: 2%	0/41	5-year: 23%	5-year: 34%
CRS + HIPEC group	30	63.9	6.8 (median, range 5.1–9.2)	9 (median; range 5–15)	CCO: 79.3% CC1 or 2: 20.7%	Grade 3 and 4: 20.7%	0/30	10.7	19.3
CRS only group	30	64.2	2.1 (median, range 1.0–5.4)	10 (mean; range 6–16)	CCO: 74.4% CC1 or 2: 25.6%	Grade 3 and 4: 20.7%	0/30	13.1	29.7

PCI: Peritoneal Cancer Index; Completeness of Cytoreduction Score (CC-S): CC-0 = no macroscopic residual cancer, CC-1 = residual nodules < 2.5 mm, CC-2 = residual nodule between 2.5 and 25 mm; CRS: Cytoreduction surgery; HIPEC: Hyperthermic Intraperitoneal Chemotherapy

Gaudet Chardonnet and al, found three factors associated to an increased OSAR: a recurrence more than 12 months after initial surgery, type 1 histologic subtype, and treatment of PC recurrence with chemotherapy. There was no difference in both groups in terms of histologic subtype or use of chemotherapy.

## Limitations

The limitations of this study are associated with its retrospective nature and the absence of randomization. The population sample size was small. This can be explained by the fact that the pathology studied is relatively rare. There is also a lack of information regards pre-operative treatments and details of complications types in the CRS + HIPEC group.

## Conclusion

The use of HIPEC combined to CRS did not have any significance as regard the DFS and OS over CRS alone in patients with primary or recurrent peritoneal metastasis of endometrial cancer. Despite a poorer prognosis in case of recurrent disease, the use of HIPEC in women with peritoneal recurrence resulted in survival rates comparable to those of a primary advanced stage managed with optimal CRS. Preoperative selection, management and evaluation of patients is recommended. There is also a need for randomized clinical trials, comparing both the medical and surgical approach, but also evaluating the HIPEC effectiveness.

## Data Availability

The datasets used and/or analysed during the current study available from the corresponding author on reasonable request.
